# Computational identification of disease models through cross-species phenotype comparison

**DOI:** 10.1242/dmm.050604

**Published:** 2024-07-01

**Authors:** Pilar Cacheiro, Diego Pava, Helen Parkinson, Maya VanZanten, Robert Wilson, Osman Gunes, the International Mouse Phenotyping Consortium, Damian Smedley

**Affiliations:** ^1^ William Harvey Research Institute, Queen Mary University of London, London, EC1M 6BQ, UK; ^2^ European Bioinformatics Institute (EMBL-EBI), Wellcome Trust Genome Campus, Hinxton, Cambridge, CB10 1SD, UK; ^3^ National Human Genome Research Institute, National Institutes of Health, Bethesda, MD 20892, USA

**Keywords:** Mendelian disorders, Cross-species phenotype comparison, Mouse models

## Abstract

The use of standardised phenotyping screens to identify abnormal phenotypes in mouse knockouts, together with the use of ontologies to describe such phenotypic features, allows the implementation of an automated and unbiased pipeline to identify new models of disease by performing phenotype comparisons across species. Using data from the International Mouse Phenotyping Consortium (IMPC), approximately half of mouse mutants are able to mimic, at least partially, the human ortholog disease phenotypes as computed by the PhenoDigm algorithm. We found the number of phenotypic abnormalities in the mouse and the corresponding Mendelian disorder, the pleiotropy and severity of the disease, and the viability and zygosity status of the mouse knockout to be associated with the ability of mouse models to recapitulate the human disorder. An analysis of the IMPC impact on disease gene discovery through a publication-tracking system revealed that the resource has been implicated in at least 109 validated rare disease–gene associations over the last decade.

**Research Simplified**

Researchers often delete or mutate specific genes in mice to investigate the roles of these genes and their protein products in human disease. The International Mouse Phenotyping Consortium (IMPC) is a global effort by multiple research institutes to generate a collection of mice with mutations in every protein-coding gene and catalogue the physiological effects (or phenotype) of the mutations. This is important as these physiological effects may mimic the symptoms of human diseases, enabling further research into these conditions.

The authors of this study analysed the IMPC phenotypic data recently released in December 2023 using the PhenoDigm algorithm, which compares the phenotypes in the mice with the phenotypes of human diseases. Out of 2400 mouse models with mutations in genes that are associated with a human disease, the algorithm found that 1311 mouse models mimicked the corresponding human disease, a twofold increase from the previous IMPC data release in March 2019. Moreover, 692 mouse models that had already been reported in 2019 showed additional phenotypes in new physiological systems. The authors also found that the IMPC resource was used in 125 published studies that identified roles of 109 genes in diseases, highlighting its importance in disease gene discovery.

Overall, this study highlights the value of the IMPC for human genetic studies, as it is important for researchers to know if the mice they are using in their laboratory truly resemble the human disease they are investigating.

## INTRODUCTION

The International Mouse Phenotyping Consortium (IMPC) has been generating functional knowledge for mouse orthologs of human genes for more than 10 years. This is a global effort of multiple academic biomedical research centres comprising mutant mouse line production, standardised and comprehensive phenotyping screens across several life stages, and computational analysis. The quality-controlled data undergoes a rigorous statistical pipeline that is made available to the scientific community through frequent data releases (DRs). For each knockout (KO) line, abnormal phenotypes are being ascertained across a range of physiological systems through comparison with wild-type lines (Kurbatova et al., 2015; Haselimashhadi et al., 2020). These gene–phenotype associations have been investigated to increase our knowledge on human disease using multiple, complementary approaches: (1) to learn about sexual dimorphism and pleiotropic traits (Karp et al., 2017; Munoz-Fuentes et al., 2022), (2) to reveal previously unidentified candidate genes for multiple physiological systems and disease types (Higgins et al., 2022; Bowl et al., 2017; Chee et al., 2023; Cacheiro et al., 2022, 2020) and (3) to investigate how well the current phenotypic screens capture specific human traits (Cacheiro et al., 2023; Lindovsky et al., 2023).

The use of controlled vocabularies that capture the phenotypic abnormalities observed in the mutant mice (Mammalian Phenotype Ontology, MP) (Smith and Eppig, 2009; Baldarelli et al., 2024) and the clinical manifestations observed in patients affected by a particular disease (Human Phenotype Ontology, HPO) (Kohler et al., 2017) enables the systematic computation of phenotypic similarities between a mouse model and a Mendelian disease. This is performed by the PhenoDigm algorithm (Smedley et al., 2013) as part of the IMPC computational pipeline and allows us to identify which mouse KOs are able to mimic, even if partially, the phenotypes described in humans. Therefore, new models of known human disease–gene associations can be revealed, including those for which no mouse model existed before. As this is calculated for all the pairwise combinations of mouse mutants (broken down into homozygous versus heterozygous mutants and embryo versus early adult stages) and rare disorders with available HPO annotations, previously unreported gene–disease links can also be uncovered, e.g. a gene not known to be associated to disease shows a high phenotypic similarity with multiple well-characterised developmental syndromes (Cacheiro et al., 2020). Such examples included mouse KOs for *Lmtk2* or *Gga1* with several reproductive system phenotypes mimicking spermatogenic failure disorders (OMIM:618152, OMIM:618429 and OMIM:618433). A previous *Lmtk2* model collated by the Mouse Genome Informatics (MGI) resource displayed abnormal male reproductive system phenotypes (Kawa et al., 2006). For *Gga1*, existing non-IMPC models showed various abnormal phenotypes affecting different physiological systems (Govero et al., 2012). The PhenoDigm algorithm also identified IMPC models of *Dazap2* displaying significant abnormal eye phenotypes (Groza et al., 2023) similar to those reported in patients affected by retinitis pigmentosa (OMIM:615565, OMIM:300155 and OMIM:268025) whereas phenotypes in KOs for *Bicdl2* (Groza et al., 2023) mirrored those present in polycystic kidney diseases (OMIM:617610, OMIM:263200 and OMIM:620056). Neither of these two genes have other mouse models with abnormal phenotypes as compiled by the MGI resource. It is important to highlight that our comparisons rely exclusively on genotype–phenotype data accessible through the MGI database due to the automated process of our PhenoDigm algorithm pipeline and that other models may have been documented in the literature and not yet captured by MGI.

The viability screen performed in all mutant lines provides invaluable information to understand the variable degree of intolerance to loss-of-function variation across the protein-coding genome. The strong association between lethal lines and Mendelian phenotypes in humans has been the foundation of multiple novel disease gene discovery approaches (Cacheiro et al., 2022, 2020). One successful strategy identified a set of genes that are essential for mouse organism development but non-essential for cell proliferation and that are highly constrained according to several metrics. Further comparison of embryo and early adult mouse phenotypes and clinical features observed in patients with variants in genes lacking a molecular diagnosis led to the prioritisation of a subset of previously unreported ‘developmental lethal’ genes, with three of them having been functionally validated as the causative gene for the disorder (Vetro et al., 2023; Cousin et al., 2021; Rodger et al., 2020).

Here, we focus on a set of 2742 genes known to be associated with rare disorders and investigate the ability of their corresponding IMPC mouse ortholog models to mimic the phenotypes observed in patients, providing several examples. We also evaluate this performance in the context of various disease and mouse model features such as genotype, number of phenotypic annotations and disease area, and highlight some of the challenges and limitations of the IMPC approach. Lastly, we provide an overview of the translational impact of this resource in rare disease research by conducting a review of the literature using the IMPC repository for gene discovery in Mendelian conditions. This analysis extends beyond the phenotypes ascertained through the IMPC pipeline to the availability of IMPC mutant lines to researchers. Together, these have allowed the generation of new knowledge that can be tracked through their use in scientific publications.

## RESULTS

### DR20.1 and update on models of rare disease

**Fig. 1.**
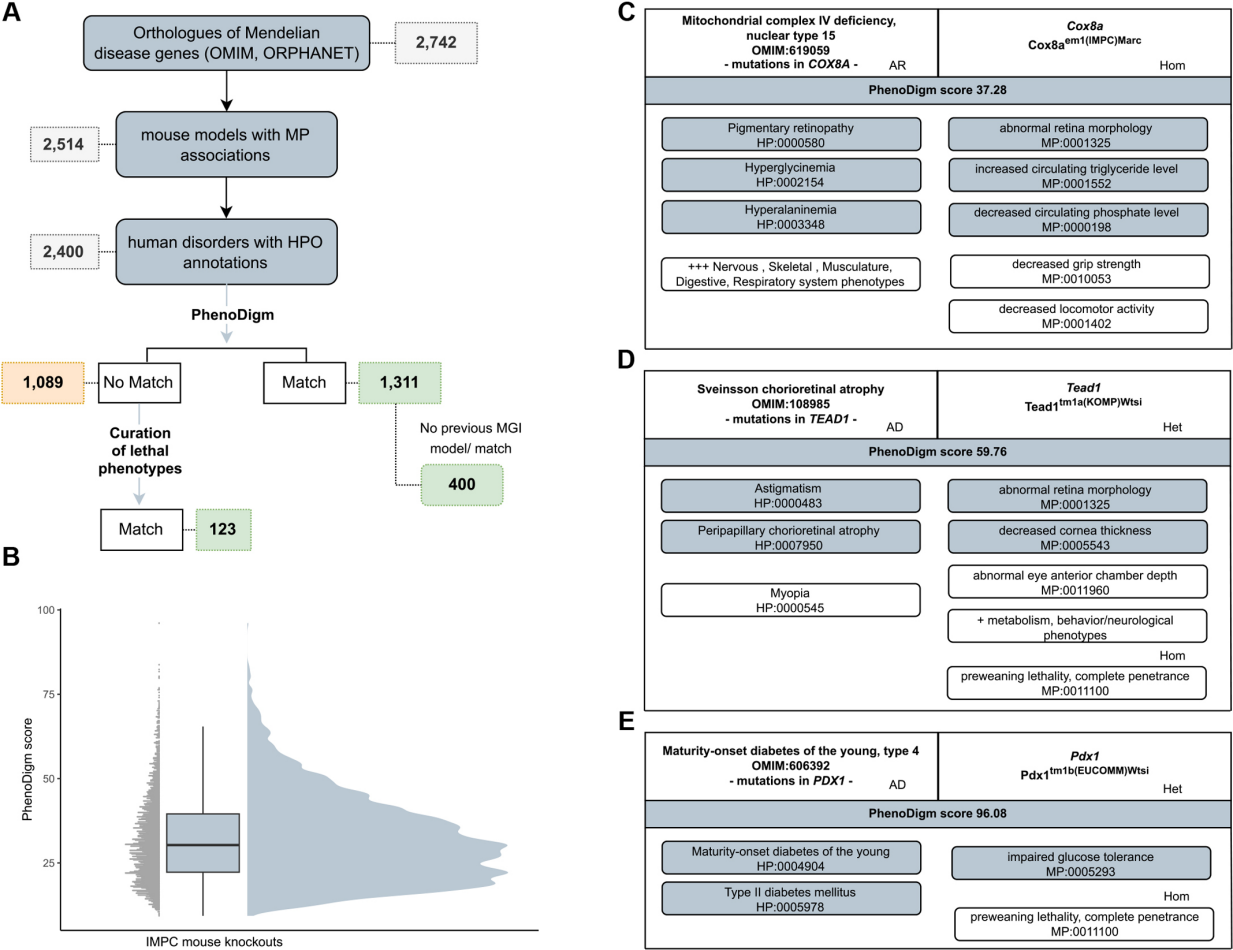
**Automated identification of disease models.** (A) Data from International Mouse Phenotyping Consortium (IMPC) data release (DR) 20.1 showing the number of mouse genes with a human ortholog associated to Mendelian disease and the subsequent number of PhenoDigm matches, where the mouse model is able to mimic, at least partially, the phenotypes observed in patients. (B) Distribution of PhenoDigm scores for the mouse model–disease pairs with a percentage score >0. Grey dots show individual data points and the blue shaded region shows the density curve. The box shows the interquartile range, the whiskers show the variability outside the interquartile range and the median is marked with a line. (C) An example illustrating the IMPC mouse abnormal phenotypes for *Cox8a* (right), the human phenotypes for the associated disorder (left), the individual ontology terms that are matched between the two organisms and the final percentage PhenoDigm score. (D) The homozygous (hom) knockout (KO) for *Tead1* shows preweaning lethality, whereas the heterozygous (het) model mimics some of the eye phenotypes observed in patients affected by the associated disorder. (E) The heterozygous KO for *Pdx1* achieves the maximum similarity score capturing the human phenotypes described for the human disease maturity-onset diabetes of the young, type 4. AD, autosomal dominant; AR, autosomal recessive; het, heterozygous; hom, homozygous; HPO, Human Phenotype Ontology; MGI, Mouse Genome Informatics; MP, Mammalian Phenotype Ontology; OMIM, Online Mendelian Inheritance in Man.

According to the most recent data release at the time of this analysis (DR20.1, December 2023), 8707 mouse genes have entered the IMPC phenotyping pipeline. For 2742 (31%), the one-to-one human ortholog is associated with a Mendelian disease according to Online Mendelian Inheritance in Man (OMIM)/Orphanet resources (Amberger et al., 2019; http://www.orpha.net). Our PhenoDigm algorithm requires MP-encoded phenotypes for the mouse KO and HPO-encoded phenotypes for the associated disease to compute the phenotypic similarity between them. Out of 2400 gene KOs meeting these criteria, 1311 (55%) are able to mimic, to some extent, the corresponding human disease phenotypes (Fig. 1A). This is a twofold increase compared to that in our previously published report based on DR10.0 (March 2019) in which PhenoDigm matches were found for 615 human disease genes in the 5861 phenotyped lines (Cacheiro et al., 2019). An additional 123 genes are matched when comparing preweaning lethality annotations in the mouse and manually curated reports of early, pre-infant death in the OMIM clinical records (Cacheiro et al., 2024). This number increases to 156 if we include death during childhood. These are gene-level results and it is important to emphasise that although multiple mouse models may exist for a given gene (e.g. embryo, early adult, heterozygous, homozygous), only one might obtain a PhenoDigm match. Similarly, among several disorders associated with the same gene, only one might achieve a PhenoDigm match, based on the documented human phenotypes. The complete list of pairwise mouse model–disease scores and matching terms is available to explore and download from https://diseasemodels.research.its.qmul.ac.uk/.

PhenoDigm generates a percentage-based score for all the pairwise MP–HPO comparisons relative to the maximum possible scores, i.e. that of a mouse perfectly mimicking the disease phenotypes. The distribution of this percentage score for the IMPC models is illustrated in Fig. 1B. An example of one PhenoDigm match is shown in Fig. 1C, where the IMPC homozygous model for *Cox8a* shows several metabolism and eye abnormalities mimicking several phenotypes described in patients affected by disorders associated with the human ortholog and with a score (37.28) close to the average (mean=32.1, s.d.=12.7). A higher score (59.76) is obtained by the *Tead1* heterozygous KO (illustrated in Fig. 1D) with several abnormal eye phenotypes. A near-maximum similarity score (96.08) is achieved by a model of maturity-onset diabetes of the young, type 4, in which the heterozygous KO-impaired glucose tolerance captures the diabetes phenotypes available as HPO terms (Fig. 1E). Detailed information on these three models is available through the IMPC resource (Groza et al., 2023).

The IMPC pipeline additionally computes the phenotypic similarity for different mouse lines and associated phenotypes available through the MGI database, a resource that collates mouse data from different sources (Martin et al., 2019; Stark et al., 2021). By comparing the scores of the separate IMPC and external MGI models (if available), we were able to identify 400 genes modelled by IMPC with no previous MGI mouse–human disease phenotypic match, i.e. no previous mouse KO captured by MGI or no existing mouse model with phenotypic overlap with the disease phenotypes as computed by PhenoDigm. For the examples illustrated in Fig. 1, (1) *Cox8a* has no other mouse model as captured in MGI, (2) previous models are reported for *Tead1* that do not achieve a positive score (Sawada et al., 2008; Chen et al., 1994) and (3) different existing models for *Pdx1* show similar overlap with the associated disease phenotypes (Holland et al., 2002, 2005). Conversely, PhenoDigm fails to get a positive score for 203 genes in the current IMPC pipeline with documented abnormal phenotype and disease association as outlined in the MGI resource.

It is important to note that not only has the number of mouse lines available through the IMPC programme significantly increased since DR10.0 in 2019 (from 5861 to 8707 phenotyped genes in DR20.1), but the number of genes associated to Mendelian disorders has also increased: 4986 OMIM/Orphanet one-to-one human orthologs of mouse genes were incorporated into the DR20.1 PhenoDigm analysis pipeline in comparison to 4166 genes available in 2019. Additionally, in DR20.1, we found additional phenotypes in previously unidentified physiological systems for 692 gene KOs that had already entered the phenotyping pipeline in DR10.0 and with a link to a human disease in OMIM/Orphanet at the time. This gradual accumulation of data for a line is explained by some features of the IMPC programme, including its multicentre nature, the generation of different mutants for the same gene (homozygous and heterozygous for lethal lines) and the phenotyping occurring at different developmental stages.

### Analysis of PhenoDigm matches

**Fig. 2.**
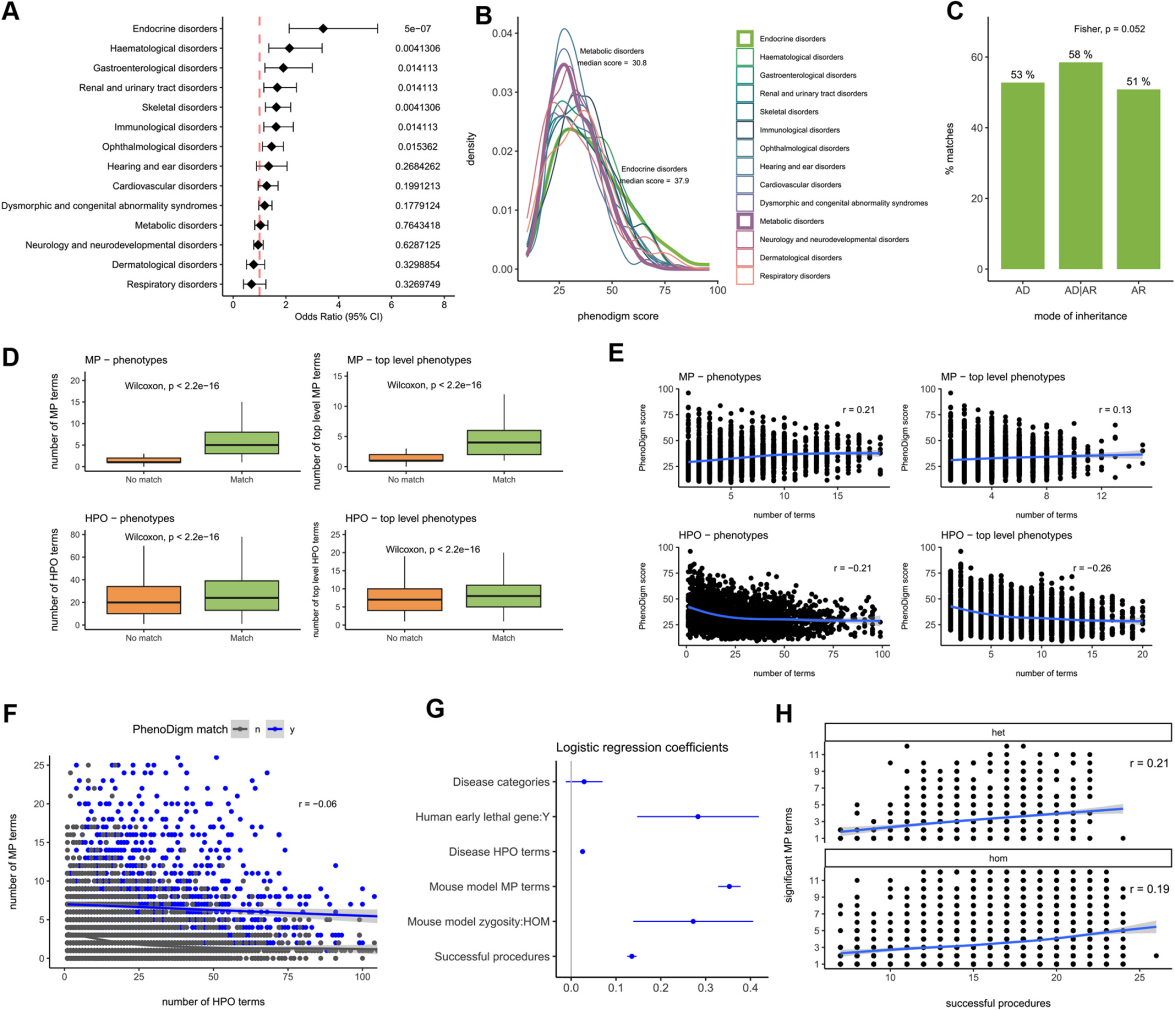
**Analysis of PhenoDigm matches.** (A) Disease categories overrepresented among PhenoDigm matches. (B) Distribution of PhenoDigm scores for the mouse model–disease pairs with a PhenoDigm match (maximum score per gene). (C) Percentages of matches by disease mode of inheritance. AD, autosomal dominant; AR, autosomal recessive. (D) Comparison of mouse and human phenotypes between PhenoDigm matches and non-matches. Boxes show the interquartile range, the whiskers show the variability outside the interquartile range and the median is marked with a line. (E) Correlation between PhenoDigm scores (PhenoDigm matches) and number of mouse and human phenotypes. (F) Correlation between number of MP terms and the number of HPO terms associated to each mouse model–disease pair for PhenoDigm matches and non-matches. (G) Coefficients from a logistic regression model fitted to describe and explain the relationship between the binary variable PhenoDigm match/non-match and several mouse model, gene and disease features. (H) Correlation between the number of phenotypic procedures completed and the number of significant MP terms for homozygous (viable) and heterozygous (homozygous lethal) early adult models (outliers removed, one phenotyping procedure can lead to several MP term associations). het, heterozygous; hom, homozygous; HPO, Human Phenotype Ontology; MP, Mammalian Phenotype Ontology. Odds ratio and Fisher's exact tests *P*-values (Benjamini–Hochberg adjusted) are shown in A; two-sided *P*-values for Fisher's exact test and Wilcoxon test are shown in C,D; Pearson's correlation coefficients (r) are shown in E,F,H; point estimates of logistic regression coefficients and 95% c.i. are shown in G. Number of mouse model–disease pair PhenoDigm matches, *n*=2621; PhenoDigm non-matches, *n*=6881. Multiple mouse models may exist for a given gene. Similarly, several disorders may be linked to the same gene.

In order to investigate which features may impact the ability of IMPC mouse KOs to recapitulate the phenotypes observed in patients, we further annotated all the OMIM/Orphanet genes that entered our PhenoDigm pipeline with a high-level disease class (based on PanelApp level 2 categories; Martin et al., 2019; Stark et al., 2021; see Materials and Methods) and the evidence for the gene–disease association (based on the OMIM category ‘disorders with molecular basis known’ and the PanelApp categorisation of green, amber and red levels). Additionally, we explored the performance of lines that are lethal versus viable in a homozygous state, and the coverage of both the HPO and MP annotations for the disease and the mouse models, respectively. When we limited the analysis to genes classified as ‘green’ (diagnostic grade), the percentage of matches slightly increased to 58% (1108/1924) compared to 55% for all associations. This percentage was not uniform across disease categories, ranging from 81% for endocrine disorders to 48% for respiratory disorders and 57% for neurology and neurodevelopmental disorders, the category with the highest number of genes. An enrichment analysis confirmed several disease classes for which phenotype matches were overrepresented. (Fig. 2A). The IMPC pipeline measures and captures significant phenotypes more in certain systems than in others, which could explain this difference; e.g. 764 IMPC genes show an endocrine/exocrine gland phenotype, 812 have a nervous system phenotype, but only 141 have a respiratory system phenotype. Importantly, 3244 genes have an associated ‘homeostasis/metabolism phenotype’, making it the category with the highest number of gene–phenotype associations. Additionally, although specific abnormalities under this parental term in the MP ontology have a perfectly matching term in the HPO, e.g. ‘MP:0005478, decreased circulating thyroxine level’ and ‘HP:0031507, decreased circulating T4 concentration’, in humans, this phenotype is found under the ‘abnormality of the endocrine system’ grouping term. Besides differences in the proportion of PhenoDigm matches across disease categories, the distribution of the scores for those genes with a match is not uniform across disease categories (Fig. 2B). Considering the mode of inheritance for the associated disorders, the proportion of matches was higher, although not statistically significant, for the genes linked to both autosomal dominant and recessive inheritance compared to those linked to just dominant or just recessive inheritance (Fig. 2C).

The number of phenotypes described both for the mouse mutant and the associated human disease is significantly different between PhenoDigm matches and non-matches (Fig. 2D). For the PhenoDigm matches, there is a positive but very weak correlation between the value of the score and the number of MP and top-level MP terms, whereas the opposite is true for the number of HPO and top-level HPO terms (Fig. 2E). This is as expected given the PhenoDigm algorithm, in which the queries are the disease–HPO annotations; so the more terms there are, the less likely it is that all the HPO annotations will be matched to give a high score. In contrast, having more MP annotations for the mouse matches increases the chance of a high score. No meaningful correlation was found between the number of MP terms and HPO terms for each mouse model–disease pair (Fig. 2F).

Focusing on mouse model features, for the gene KOs with viability assessment results, we found a significant association between viability category and the likelihood of obtaining a phenotype match (*P*=0.008), with the percentages of positive scores being 50, 52 and 58% for the sub-viable, lethal and viable categories, respectively. The percentage of genes in each category for all the lines with viability data are 8, 24 and 68%, respectively, a ratio that has remained fairly constant across data releases. However, when we consider the subset of disease-associated genes irrespective of the PhenoDigm match outcome, the percentages (12, 38 and 50%, respectively) illustrate the previously reported enrichment for mouse embryonic or early postnatal lethality among Mendelian genes (Cacheiro et al., 2020; Dickinson et al., 2016; Georgi et al., 2013).

A logistic regression model was fitted using these and other mouse model-, human gene- and disease-specific features, in which PhenoDigm matches (class 1) correspond to the mouse model–disease pairs with PhenoDigm >0 and PhenoDigm non-matches (class 0) correspond to pairs with no HPO–MP match. The regression coefficients shown in Fig. 2G highlight the effect of the number of phenotyping procedures completed (phenotyping for some lines has not yet been concluded) and number of significant mouse phenotypes and HPO terms associated with the human disorder*.* Additionally, the genes belonging to different disease categories and those for which early death has been reported are more likely to obtain a match*.* Consistent with the positive direction of the association for these features, the genes in the latter category correlate with those associated with multisystemic phenotypes (Cacheiro et al., 2024). The positive effect of homozygous KO models is in agreement with a higher proportion of matches found among the viable lines described above. Homozygous, viable models showing a higher number of significant phenotypes and being more likely to obtain a match are also associated with a higher number of successful phenotyping procedures completed (*P*<2.2×10^−16^). However, the correlation between the number of procedures completed and significant MP terms is again weak (Fig. 2H), suggesting that other factors may be involved (see Discussion).

The most frequent disease categories among the PhenoDigm matches ‘rescued’ by manual comparison of lethal phenotypes between mouse and human include neurology and neurodevelopmental disorders, followed by metabolic disorders, dysmorphic and congenital abnormality syndromes, and cardiovascular disorders. In this particular context, skeletal disorders, while being the third most prevalent category among all pre-infant lethal phenotypes (Cacheiro et al., 2024), exhibit lower prevalence within this specific subset of genes associated with early lethal phenotypes in humans for which the mouse KOs are not able to capture other disease phenotypes.

### Impact of IMPC on Mendelian gene discovery

Through a publication-tracker system and subsequent manual curation to ascertain the distinct use of the resource (see Materials and Methods), we identified 7111 publications from 2011 to 2024 matching at least one of these criteria: (1) IMPC publication; (2) the publication uses data generated by the IMPC or cites an IMPC publication; or (3) uses embryonic stem cell/mouse KO lines generated by the IMPC.

**Fig. 3.**
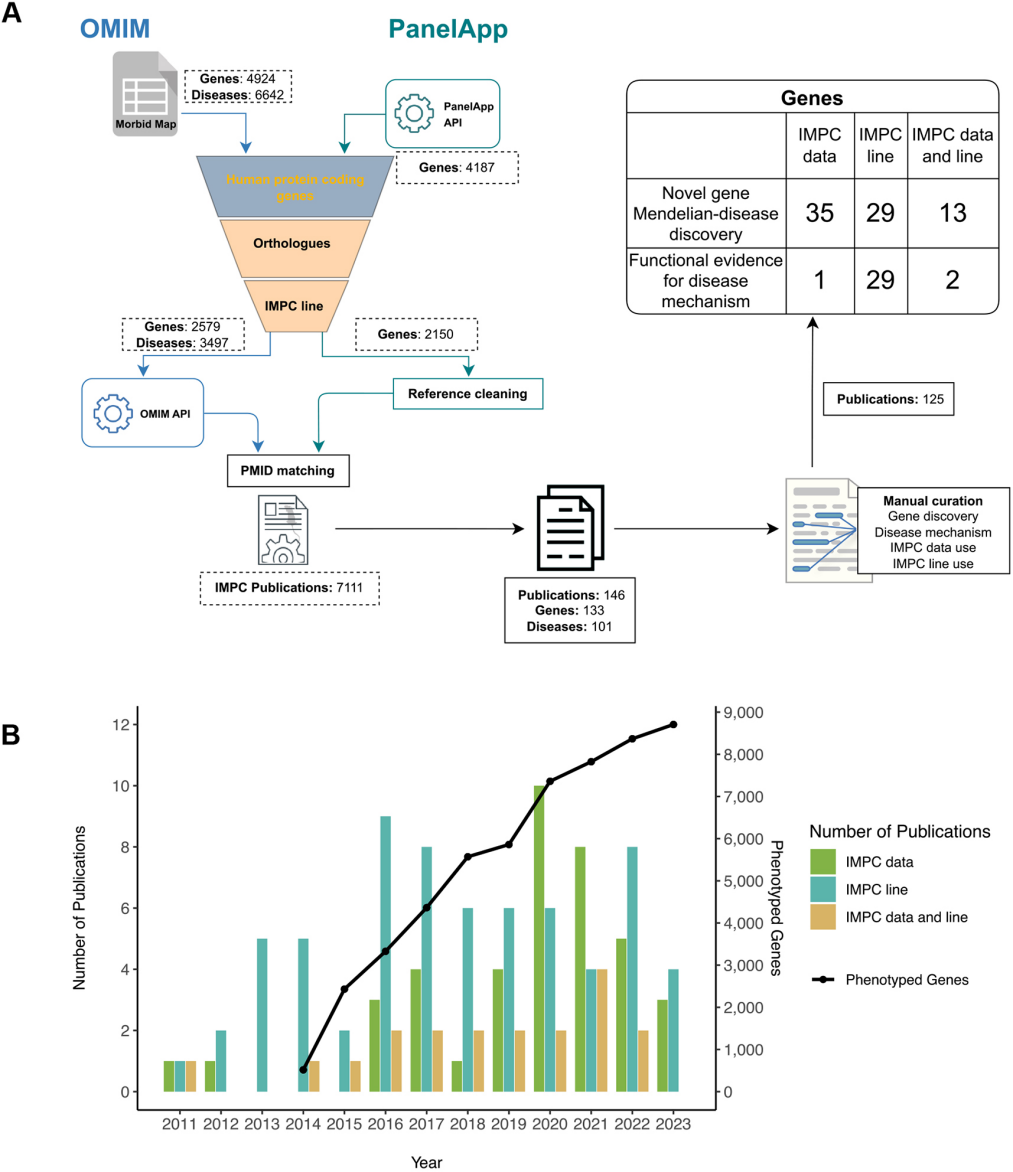
**Impact on Mendelian gene discovery.** (A) Process for gene and disease selection to examine the impact of the International Mouse Phenotyping Consortium (IMPC) on Mendelian gene discovery. Genes from OMIM and Panel App were selected based on being human protein-coding genes (HUGO Gene Nomenclature Committee, HGNC), having a mouse ortholog and being part of the IMPC phenotyping line. Associated diseases to genes listed in OMIM were selected for information retrieval. The PMIDs of publications used within OMIM and PanelApp for each gene/disease were cross-referenced against PMIDs from IMPC-related publications. Papers that contributed evidence to establish previously unreported gene–disease discoveries or functional evidence of a disease mechanism were manually curated. Within that cohort, the number of genes that were investigated using IMPC phenotyping data and/or an IMPC line were classified. (B) Number of IMPC publications that contributed evidence on gene discoveries per year and number of phenotyped genes by the IMPC.

To assess the impact of the IMPC on Mendelian gene discovery, we investigated which of these IMPC-related publications have described a previously unreported gene discovery. To this end, we extracted the PubMed reference number (PMID) from the curated IMPC publications and cross-referenced them with PMIDs used as evidence for genes and associated diseases within OMIM (Amberger et al., 2019) and genes in PanelApp (Martin et al., 2019; Stark et al., 2021) for the genes with a mouse ortholog that has entered the IMPC pipeline. The process for gene and disease selection is elucidated in Fig. 3A and described in the Materials and Methods. These publications were then manually curated to establish the following categories: (1) previously unreported gene–Mendelian disease discovery publications and (2) publications providing relevant functional evidence for understanding mechanisms. Each category is further broken down into: (1) publications that use IMPC data, i.e. gene–phenotype associations assessed by the IMPC phenotyping pipeline and (2) publications that use IMPC lines (embryonic stem cells or mice).

From 125 publications in total, the IMPC resource was directly or indirectly involved in establishing 109 previously unreported gene–disease associations. For previously unreported Mendelian disease gene discoveries, 35 genes were established from IMPC data, 29 from embryonic stem cell/mouse lines and 13 from both data and lines. As for describing previously unknown functional evidence for a disease mechanism, one gene was established from IMPC data, 29 from IMPC lines and two from both (Fig. 3A). Interestingly, we found IMPC data/lines were involved in Mendelian disease gene discovery or functional disease mechanisms for an additional set of 26 genes categorised as amber or red by PanelApp (see Materials and Methods). The IMPC has generated more and more data since its inception in 2011 as reflected in Fig. 3B. As an example of IMPC data supporting disease gene discovery, the role of biallelic variants in *KDELR2* causing a form of osteogenesis imperfecta with neurodevelopmental features was supported by incompletely penetrant preweaning lethality, bone structural abnormalities in early adult mice, and decreased size, abnormalities in head shape and size, facial dysmorphology, and abnormal embryonic body wall structure reported in the embryo in the IMPC mutant (Efthymiou et al., 2021).

## DISCUSSION

Comparison of phenotypes across species enables the automated identification of mouse models that recapitulate the phenotypes of the Mendelian disorder associated with the corresponding human ortholog. The standardised, multisystemic, unbiased and high-throughput nature of the IMPC phenotyping pipeline generates a dataset that can be investigated to identify factors associated with the ability of the mouse models to phenocopy human disease. We found that for almost half of the mouse orthologs of human disease genes that the IMPC has tested, there was no phenotypic overlap detected by our algorithm. Manual curation of lethal phenotypes reported in humans increased the percentage of matches, suggesting that the current approach is not well designed to compare these age of death records. Some of the constraints include ambiguous reports of age of death and the difficulty in extracting these from OMIM clinical records, the limited number of current HPO annotations regarding lethality in humans, and the fact that genes with prenatal lethal phenotypes are likely underrepresented in current disease databases (Cacheiro et al., 2022; Dawes et al., 2019). Efforts are being made to improve and expand prenatal terms in humans including prenatal and perinatal death (Dhombres et al., 2022). This, together with the increase in the number of prenatal sequencing studies, will potentially improve the performance for KO models with embryonic phenotypes. Improvements to the algorithm could be implemented to align phenotypes between mice and humans for the same disorder and gene at equivalent developmental stages.

The differences in performance between viable and lethal lines highlights several aspects that should be taken into consideration: (1) although we are evaluating one model (homozygous KO early adult) for viable lines, for lethal lines, we are, in most cases, separately evaluating a homozygous mouse embryo model and a heterozygous KO early adult model against a single set of human disease-associated phenotypes; (2) in the lethal homozygous KO models, we are assessing the complete loss of function of a gene, whereas for heterozygous mouse models with viable phenotypes, we are looking at a haploinsufficiency mechanism. Previous evidence suggests that for embryonic lethal lines, heterozygous mice are often able to mimic phenotypes observed in humans carrying biallelic variants, suggesting hypomorphic effects of protein-truncating variants (Blackburn et al., 2020; Beecroft et al., 2021). To add to this challenge, the IMPC KOs do not necessarily model the type of variants observed in humans, e.g. missense variants and/or gain-of-function mechanisms in different types of disorders (Foreman et al., 2023; Gallego-Martinez et al., 2019; Martinez-Barrios et al., 2022), although precision modelling of such variants has been performed in mice in other projects.

Other limitations pertaining to the IMPC phenotyping and statistical analysis pipeline involve the heterogeneous implementation of certain phenotypic assays across phenotyping centres, which restricts the ability to capture human phenotypes. This has been discussed in the specific case of congenital heart disorders (Cacheiro et al., 2023). In addition, the human phenotypes described for a disorder vary in frequency and come from case reports in the literature, whereas an associated phenotype as per the IMPC pipeline is only assessed after strict quality-control procedures and statistical significance for the KO compared to a set of matched wild-type mice (Haselimashhadi et al., 2020). Hence, the inability to identify relevant abnormal phenotypes in mice, which are observed in humans, does not necessarily imply that the phenotypes are not present. It is possible that the assay required to capture the relevant phenotype was not conducted or that the sample size was not sufficient to detect the abnormal phenotype, as only large effects are likely to be detected.

Factors impacting the ability of mouse KOs to model human disease beyond the phenotyping pipeline, statistical analysis and cross-species comparison algorithm have been previously discussed and include the limitation of using one uniform genetic background and potentially different phenotype associations for single gene KOs in different backgrounds (Simon et al., 2013; Doran et al., 2016). Moreover, the more heterogeneous background found in humans, with polygenic factors contributing to variable expressivity and penetrance (Oetjens et al., 2019; Fahed et al., 2020) and differences in developmental gene expression profiles between the two species that could translate into phenotypic differences (Cardoso-Moreira et al., 2020) should also be considered.

Overall, the proportion of PhenoDigm matches, computed using the exact same criteria and sources of human disease annotations, has increased from a previously published report (Cacheiro et al., 2019). Given the nature of the IMPC approach, we expect this percentage to keep rising, as all the phenotypic assays for lines that have already entered the pipeline are completed and analysed. Importantly, for many human disease genes, the IMPC mouse ortholog models constitute either the first mouse model or a model achieving a higher similarity score. One unique feature of the IMPC is the systematic annotation of negative phenotypes, where assays have not recorded a statistically significant abnormal phenotype, and, in the future, we plan to incorporate these data into the algorithm to improve accuracy. In addition, all human disease phenotypes are currently treated equally, but recently, frequencies have been added (how often patients with the disease exhibit the phenotypes), and these will also be incorporated to improve performance.

As mentioned earlier, our automated pipeline relies on the availability of HPO-encoded phenotypes for the human disorder. However, it is important to recognise that the set of human gene–disease associations used in our analysis is not exclusive and our approach may overlook some relevant associations. For instance, our current implementation fails to include 195 genes in the existing IMPC pipeline for which documented disease associations exist according to the MGI resource (Blake et al., 2021), or 106 diagnostic grade genes listed in PanelApp (Stark et al., 2021). Multiple sources document evidence on Mendelian phenotype associations, relying on expert curation and with varying degrees of diagnostic relevance (Robin-Tobias et al., 2024 preprint). The addition of other disorders catalogued by the Disease Ontology (Baron et al., 2024) or genes labelled as ‘amber’ and ‘red’ in PanelApp, for which the phenotype association needs further evidence and investigation, could be explored.

In addition to the identification of models for known gene–disease associations, the impact of the IMPC programme on Mendelian gene discovery can also be assessed directly through the use of the data provided by the internal pipeline or indirectly through the external phenotyping of models using embryonic stem cell lines and mouse lines generated by the Consortium. We found 125 publications describing previously unreported gene–disease discoveries and/or providing relevant evidence for the disease mechanisms for 109 genes that made use of the IMPC resource and that are captured by OMIM and/or PanelApp. Recent publications not yet collated by these resources or those not indexed in PubMed are excluded in this analysis.

In summary, this analysis highlights how the use of standardised vocabularies of phenotypic abnormalities and algorithms for cross-species comparisons facilitates the automated and unbiased identification of mouse models of disease. Here, we limited our approach to known gene–disease associations; however, the PhenoDigm algorithm may also be used to uncover previously unreported gene–phenotype links. The data generated by the IMPC constitute a valuable resource for human genetic studies, having been implicated in multiple Mendelian gene discovery studies and providing functional evidence for disease mechanisms.

## MATERIALS AND METHODS

### Automated identification of disease models – PhenoDigm pipeline and analysis of mouse model–disease matches

The PhenoDigm algorithm was used to compute the phenotypic similarity between mouse models and known human disorders (Kohler et al., 2017). The percentage-based score used here is obtained by comparing the best and mean scores for all individual pairwise HPO–MP term comparisons relative to the maximum possible score, i.e. a mouse model perfectly mimicking all the human disease phenotypes. For the purpose of our analysis, a PhenoDigm percentage score greater than 0, which implies at least one HPO–MP match, was considered a PhenoDigm match.

IMPC mouse model and phenotype associations, and information on procedures completed according to DR20.1 are available online (https://ftp.ebi.ac.uk/pub/databases/impc/all-data-releases/release-20.1/results/; data accessed on 4 April 2023). Additional MGI models (Blake et al., 2021; Eppig et al., 2017) and their associated phenotypes were also used in the analysis pipeline to identify novel/improved models [https://www.informatics.jax.org/downloads/reports/index.html (MGI_PhenoGenoMP.rpt, MGI_Geno_DiseaseDO.rpt, MGI_DO.rpt); data accessed 4 April 2023]. Human gene–disease associations from OMIM (Amberger et al., 2019) and Orphanet (http://www.orpha.net) and their associated phenotypes were obtained from the HPO (Kohler et al., 2017) [https://www.orphadata.com (en_product1.xml, en_product6.xml); https://www.omim.org/ (morbidmap.txt); https://hpo.jax.org/app/data/ontology; https://hpo.jax.org/app/data/annotations (hpo.obo); data accessed 10 November 2023]. The initial PhenoDigm pipeline relies on gene–gene ortholog mapping from Ensembl BioMart (Martin et al., 2023). A stricter one-to-one mouse–human ortholog relationship was considered for this analysis, with a mapping file being generated using data from MGI (Blake et al., 2021), Human Genome Organisation (HUGO) Gene Nomenclature Committee (HGNC) (Seal et al., 2023) and HGNC Comparison of Orthology Predictions (HCOP) (Yates et al., 2021). A one-to-one constraint was applied in both directions (human-to-mouse direction and mouse-to-human direction) and a threshold for agreement between the prediction services on what constitutes an ortholog of five or more of the 12 prediction services listed in HCOP was used. The mapping file used for these analyses is provided as Dataset 1 (data accessed 12 April 2024). Disease categories were obtained from PanelApp, a resource of expert curated gene panels for Mendelian disorders (Martin et al., 2019; Stark et al., 2021) (https://panelapp.agha.umccr.org; data accessed 5 April 2024). Gene panels were categorised in hierarchical disease categories; level 2 panel information was used in this analysis. Information on lethal phenotypes in humans was curated from OMIM clinical records and obtained from the Lethal Phenotypes Portal (Cacheiro et al., 2024) (https://lethalphenotypes.research.its.qmul.ac.uk/; data accessed 12 April 2024).

All IMPC phenotypic data, information on standardised phenotyping protocols, analysis pipelines described in this paper and Animal Research: Reporting of *In Vivo* Experiments (ARRIVE) guidelines are publicly available through the IMPC portal (https://www.mousephenotype.org/) and the FTP repository (http://ftp.ebi.ac.uk/pub/databases/impc/).

The mouse model–human disease PhenoDigm scores are available through the IMPC Disease Models Portal (https://diseasemodels.research.its.qmul.ac.uk/). Additional PhenoDigm scores corresponding to the similarity between mouse models of non-disease associated genes and known disorders pairs are provided to assist researchers in previously unidentified Mendelian gene discovery and prioritising candidates for new gene–phenotype associations in humans.

Ortholog mapping files are generated on a weekly basis as part of the internal IMPC pipeline to track the production and phenotyping of mutant mice in the Genome Targeting Repository (GenTaR). These can be accessed through the following endpoints: one-to-one ortholog calls, https://www.gentar.org/orthology-api/api/ortholog/one_to_one/impc/write_to_tsv_file; all ortholog calls (one-to-one and one-to-many), https://www.gentar.org/orthology-api/api/ortholog/write_to_tsv_file. The file used in this paper is available as Dataset 1.

The code is available at https://github.com/whri-phenogenomics/disease_models.

### IMPC publication tracking and identification of relevant papers

IMPC mines the scientific literature using natural language processing methods to generate a corpus of papers that is then reviewed by an annotator using an IMPC-specific curation/literature-tracking tool. The corpus identification is optimised for recall to avoid missing papers and uses a dictionary of search terms, including official mouse allele nomenclature that uniquely identifies each IMPC line, references to repository identifiers in mouse stock centres, portal URLs, the Knockout Mouse Project (KOMP), IMPC, and also includes papers that cite IMPC papers. The results are reviewed and categorised into papers that use IMPC datasets, methods (for example, BioConductor analysis packages) and mouse lines. Authors do not always use the official nomenclature; therefore, annotators review the evidence in the paper to determine whether an IMPC line has been used when official nomenclature is absent. The dataset comprising reviewed papers was mined to extract papers referring to IMPC KO lines relevant to this paper that were published during 2011-2024 (https://www.mousephenotype.org/data/publications; data accessed 10 April 2024).

### Impact of IMPC in Mendelian gene discovery

To identify which of the publications identified through the publication-tracking system described in the previous section report previously unreported gene–Mendelian disease associations, we proceeded as follows. First, we selected the set of human protein-coding genes in HGNC (Seal et al., 2023) [https://www.genenames.org/ (gene_with_protein_product.txt); data accessed 9 April 2024] with a mouse ortholog that has entered the IMPC phenotyping pipeline as per DR20.1. Second, we retrieved the HGNC ID and reference list for each gene and their associated linked disease/phenotype as per OMIM Morbid Map. Using the OMIM application programming interface (API) (data accessed 15 April 2024), PMIDs within the evidence lists were used for analysis, ignoring evidence without a PMID. Subsequently, the PMIDs of the curated genes were matched with those from IMPC publications, and a similar approach was applied to the diseases. Publications were selected if a gene–disease or disease-only association was suggested. A similar process was followed to identify gene discovery papers based on genes registered in PanelApp. However, we did not extract exact data on the exact disease associated with the gene. We retrieved the complete, up-to-date gene set using the PanelApp API (https://panelapp.agha.umccr.org; data accessed 5 April 2024) and extracted the HGNC ID and publication data associated with each gene categorised as ‘green’. These are diagnostic-grade genes, with strong clinical evidence that are used in analysis and reporting within a diagnostic setting. For ‘amber’ and ‘red’ genes, the evidence is either not conclusive or low. The publication data of this gene list were refined to retain the available PMIDs. In instances where PMIDs were not directly available, the available data such as the citation details of the paper, PubMed Central reference number, linked publication websites or titles were manually curated to determine a PMID. Finally, the gene PMIDs were associated with those of the IMPC publications. After manually curating the publication list from both sources, the reported genes were categorised as ‘gene discovery’ or ‘functional disease mechanisms’. When a gene was categorised as both ‘gene discovery’ or ‘functional mechanism’, it was prioritised as ‘gene discovery’ to avoid overlap.

### Statistical analysis and software

Two-sided *P*-values for the test of independence were computed using Fisher's exact test. The Wilcoxon test was used to compare the number of HPO and MP terms between PhenoDigm matches and non-matches. Pearson's coefficient was used to compute the linear correlations. Odds ratio and the corresponding confidence intervals were obtained from a 2×2 table with the number of matches and non-matches for genes belonging to a given disease category compared to genes in any other disease class. Benjamini–Hochberg correction for multiple testing was applied. The statistical analysis described above were performed in R (https://www.r-project.org/). Logistic regression model was fitted using ‘tidymodels’ (https://www.tidymodels.org). The web application was also built using the R programming language, including the following packages: ‘shiny’ (https://cran.r-project.org/web/packages/shiny/), ‘shinydashboard’ (https://cran.r-project.org/web/packages/shinydashboard/) and ‘DT’ (https://cran.r-project.org/web/packages/DT/).

The ARRIVE guidelines are applied to the IMPC resource. The full list, including information on study design, sample size, statistical methods, experimental animals and procedures and reporting of results, is available at https://www.mousephenotype.org/about-impc/animal-welfare/arrive-guidelines/.

### Ethics declaration

This paper exclusively used openly available mouse and human data as described in the Materials and Methods. The information on ethical review committees and approval for the IMPC is available at https://www.mousephenotype.org/about-impc/animal-welfare/arrive-guidelines/ and https://www.mousephenotype.org/wp-content/uploads/2020/02/EthicalInfo2014.pdf.

## Supplementary Material

10.1242/dmm.050604_sup1 Supplementary information
