# Supplementary material for: Computational identification of disease models through cross-species phenotype comparison
Source: Dis Model Mech. 2024 Jul 1;17(6):dmm050604. doi: 10.1242/dmm.050604 (PMC11247498; doi:10.1242/dmm.050604)
Supplement: Supplementary information [file dmm-17-050604-s1.pdf]

Dataset 1. Human-mouse ortholog mapping file.

Available for download at
https://journals.biologists.com/dmm/article-lookup/doi/10.1242/dmm.050604#supplementary-data
